# Optimizing Nursing Communication for Symptom Management in Hemodialysis: Development of an Artificial Intelligence–Based Web Predictive Model for Burden Classification and Evidence Navigation

**DOI:** 10.1155/jonm/4579091

**Published:** 2025-10-29

**Authors:** Xutong Zheng, Aiping Wang

**Affiliations:** Department of Public Service, The First Affiliated Hospital of China Medical University, Shenyang, China

**Keywords:** chronic kidney disease, hemodialysis, machine learning, predictive modeling, symptom burden, XGBoost

## Abstract

**Background:**

Chronic kidney disease (CKD) is a global health concern, with hemodialysis (HD) being a vital life-sustaining treatment for affected patients. Symptom burden in HD patients significantly impacts quality of life and clinical outcomes. However, symptom management remains inadequate, especially in resource-constrained settings.

**Aim:**

This study aims to develop a predictive model to categorize symptom burden and optimize nursing interventions using machine learning.

**Design:**

A nationwide, cross-sectional study employing nonprobability convenience and multiregional sampling.

**Methods:**

Data were collected in five provinces in China: Liaoning (Northeast China), Fujian (Southeast China), Yunnan (Southwest China), Jiangsu (Eastern China), and Shaanxi (Central China). A total of 1866 HD patients were finally included. Machine learning algorithms, including elastic net regression, Boruta feature selection, and 8 classifiers, were used to develop and validate a predictive model for classifying symptom burden categories (mild vs. severe). The model's performance was evaluated using metrics such as accuracy, sensitivity, specificity, and AUC. Decision curve analysis (DCA) and SHapley Additive exPlanations (SHAP) values were employed for clinical utility and interpretability.

**Results:**

The XGBoost model demonstrated excellent predictive accuracy with an AUC of 0.994 on test data, outperforming other models. Key predictors included uremia toxin, electrolyte imbalance, and psychological symptoms. The model achieved strong calibration and high clinical utility, as confirmed by DCA. It also offered a practical tool for targeted interventions, reducing nursing workload while ensuring efficient care allocation.

**Conclusion:**

This study demonstrates the potential of machine learning models to improve symptom burden classification in HD patients. The XGBoost model, utilizing a limited set of key predictors, offers high predictive accuracy and clinical utility, providing a scalable solution for resource-constrained healthcare settings. This approach supports personalized symptom management, contributing to improved patient outcomes and efficient resource use in nephrology nursing.

**Summary**

• What Is Already Known
◦ End-stage renal disease (ESRD) significantly affect quality of life, with hemodialysis (HD) patients suffering from high symptom burden.
◦ Current symptom management strategies in HD care remain largely suboptimal and resource-dependent.
◦ Machine learning approaches have shown promise in improving patient outcomes, but their application in HD symptom classification remains underexplored.
• What This Paper Adds
◦ The XGBoost model demonstrates outstanding predictive performance with an AUC of 0.994, achieving high accuracy in classifying HD patients by symptom burden category.
◦ The model uses only eight key predictors, which enhances its clinical utility by simplifying data collection and preliminary navigation, thus reducing the nursing staff's workload.
◦ Key predictors such as uremia toxin levels, electrolyte imbalance, and psychological symptoms were identified, providing insights for targeted nursing interventions.

## 1. Introduction

Chronic kidney disease (CKD) represents a significant global health challenge, with HD serving as a life-sustaining treatment for millions of patients with ESRD worldwide [1, 2]. The prevalence of patients requiring HD has steadily increased across diverse geographical regions, with marked disparities in access and quality of care between high-income and low-to-middle-income countries (LMICs) [3, 4]. HD patients experience a complex constellation of symptoms that significantly impact their quality of life, functional status, and clinical outcomes [5–8]. Despite advancements in dialysis technology, symptom management remains suboptimal, particularly in resource-constrained settings where specialized nephrology nursing care is limited. The burden of these symptoms extends beyond physical discomfort to encompass psychological distress, social isolation, and reduced capacity for activities of daily living, highlighting the multidimensional nature of symptom experiences among this vulnerable population [9, 10].

The best symptom management for HD patients is hampered by the global shortage of nephrology nurses, particularly in LMICs [11, 12]. The difficulty of symptom assessment, which takes a lot of time and experience, exacerbates this shortage. The nurse-to-patient ratio is inadequate for thorough symptom management in both high-income and resource-constrained nations [11, 12]. By allowing for targeted interventions for patients with severe symptoms and appropriate monitoring for those with milder symptoms, the identification of symptom burden categories may aid in the optimization of resource allocation and enhance the effectiveness of care delivery.

Current studies on symptom burden in HD patients have the following limitations: (1) Lack of combination of variable-centered and individual-centered approaches. Previous systematic evaluation results showed that most studies only used variable-centered methods (such as principal component analysis and factor analysis) [13–15], which would only limit the understanding of symptoms to the whole, and could not achieve accurate identification and intervention of patients [16], or an individual-centered approach (latent category analysis) [17, 18], which leads to a dimensional disaster and increases the complexity of symptom interpretation. (2) Although there are studies that classify the symptom burden of HD patients into interpretable categories (such as high, moderate, and low symptom burden), there is a lack of follow-up predictive studies, which leads to its clinical application. (3) Most of the conclusions come from the lack of large representative samples—the sample size of most studies is even less than 300, which greatly weakens the statistical power and our confidence in the conclusions [16].

By increasing the effectiveness and precision of symptom burden classification in HD patients, machine learning techniques present novel opportunities to improve nursing assessment. The standardized tools used in traditional nursing assessment methods may not adequately capture the intricate relationships between various symptoms and contributing factors. In multidimensional data, machine learning algorithms can spot subtle patterns that traditional statistical methods might miss [19, 20]. These computational techniques could revolutionize workflow optimization in nephrology nursing practice by making it possible to quickly distinguish between patients who might benefit from more routine monitoring and those who need intensive symptom management. Given these potential benefits, exploring how various machine learning algorithms perform in categorizing symptom burden becomes an essential question for advancing evidence-based nephrology nursing practice. To the best of our knowledge, no predictive model currently exists to classify symptom burden in HD care.

The integration of variable-centered and person-centered methods offers a new framework for categorizing symptom burden in HD patients. This aligns with the Symptom Science Model 2.0, benefiting nursing practice by enabling more targeted decision-making on assessment, interventions, and resource allocation. The integration raises key questions about combining dimensional reduction and classification methods for clinically meaningful symptom burden categories. This work offers nephrology nurses a quick assessment tool by developing and contrasting machine learning models to predict symptom burden categories in HD patients by developing and validating a predictive model [21]. The instrument seeks to maximize nursing efficiency, allocate resources most effectively, and solve workforce shortages by grouping patients into mild or severe symptom categories. It provides a data-driven decision support system to help to control symptoms and applies the Symptom Science Model 2.0 to HD treatment. This study improves individualized treatment and supports fair management in several healthcare environments, improving patient outcomes and resource economy.

## 2. Methods

**Figure 1:**
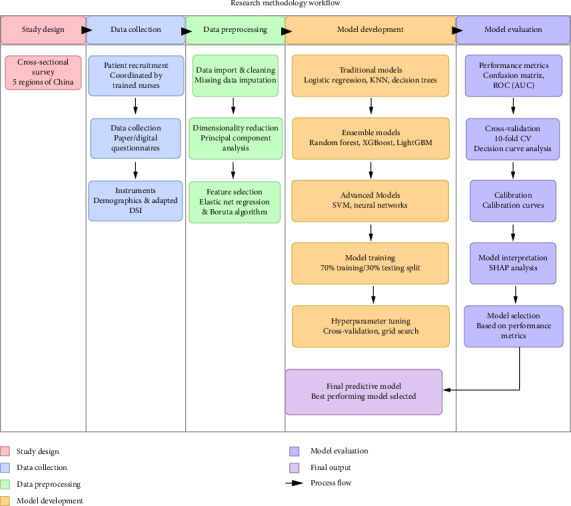
Flowchart of the study methodology for developing and validating the predictive model. The diagram outlines the key stages: participant recruitment and data collection across five Chinese provinces, data preprocessing and feature selection using elastic net and Boruta algorithms, model development with eight machine learning classifiers, and model evaluation, interpretation, and deployment.

The whole process of developing the predictive model could be found in Figure 1.

### 2.1. Study Design and Study Setting

This observational cross-sectional investigation was conducted. A convenience sampling method was implemented, supplemented by a stratified multiregional sampling strategy to enhance geographical representativity. Urban centers were strategically selected from five geographic divisions: three municipalities in Liaoning Province (Northeast China), three coastal cities in Fujian Province (Southeast China), one inland city in Yunnan Province (Southwest China), two prefecture-level cities in Jiangsu Province (Eastern China), and two industrial hubs in Shaanxi Province (Central China). Data collection was systematically conducted over a 3-month period spanning August to October 2024. The whole conducting and reporting process followed the Transparent Reporting of a multivariable prediction model for Individual Prognosis or Diagnosis (TRIPOD) + AI checklist (Supporting file 1).

### 2.2. Eligibility Criteria

The eligibility criteria could be found in Table 1.

### 2.3. Patient Recruitment and Data Collection

Trained research personnel (registered nurses or clinical interns) at participating centers facilitated participant enrollment following standardized operating procedures. Prior to implementation, all personnel participated in standardized training sessions conducted through virtual conferencing platforms to ensure protocol adherence and inter-rater reliability.

To establish content validity, a pilot validation study involving 30 demographically diverse participants was conducted, with iterative refinements made to survey instruments based on feedback (e.g., simplification of clinical terminology and optimization of response scaling) [22]. Structured data collection sessions were administered through individualized consultations, during which researchers thoroughly explained study objectives, temporal commitments, and procedural requirements.

Consenting participants were provided with two administration modalities: (1) paper-based questionnaires with optical mark recognition fields or (2) electronic QR codes linked to web-based survey instruments hosted on the Questionnaire Star platform. Research personnel maintained real-time availability to address participant inquiries while maintaining strict neutrality to prevent response bias. For participants lacking digital literacy or smartphone access, paper-based administration was prioritized, with coordinators performing immediate quality assurance checks to identify and rectify incomplete or inconsistent responses. All collected data were systematically encrypted and implemented centralized data management protocols through the Questionnaire Star digital platform.

### 2.4. Ethical Consideration

The study protocol received formal ethical clearance (Approval ID: 2024-633) from the Institutional Review Board of China Medical University's First Affiliated Hospital, which served as the central ethics committee for this multicenter study. All participating clinical sites across the data collection regions relied upon and formally accepted this approval for their involvement in the study, in accordance with national regulations on ethical review for multicenter research. The study was conducted in full compliance with the Declaration of Helsinki. Voluntary participation was strictly maintained throughout the research process, with explicit guarantees of participant autonomy regarding withdrawal rights at any investigation stage. All analytical processes incorporated robust data anonymization protocols and encryption safeguards to preserve participant confidentiality and ensure compliance with international data protection standards.

### 2.5. Instruments

#### 2.5.1. Demographic Information

Demographic information we collected including gender, age, education level, minority group, marriage, residence, solitude, average income, protopathy, medical insurance, working condition, dialysis duration, and dialysis time week.

#### 2.5.2. Adapted Dialysis Symptom Index (DSI)

The DSI, originally developed by Weisbord et al., (2004) at the University of Pittsburgh, is a multidimensional instrument designed to evaluate symptom burden in HD populations. Its initial configuration comprised 25 somatic manifestations (e.g., insomnia, fatigue, anorexia, and gastrointestinal disturbances) and 5 psychological domains (e.g., anxiety and emotional distress). Subsequent modifications by Hao Yanhua enhanced the instrument's granularity through the incorporation of symptom frequency and severity metrics.

The adapted DSI operationalizes symptom assessment across four psychometric domains: (1) presence/absence (dichotomous scoring), (2) frequency (ordinal scale), (3) intensity (ordinal scale), and (4) perceived burden (ordinal scale). While symptom occurrence employs a binary scoring protocol (0 = *absent*, 1 = *present*), the remaining dimensions utilize a validated 5-point Likert scaling system (1 = *minimal*, 5 = *extreme*). Psychometric evaluations of this modified instrument demonstrate robust internal consistency, with a Cronbach's α coefficient of 0.939–0.958 confirming scale reliability in prior clinical implementations [23].

### 2.6. Calculation of the Outcome

Based on the results of the previous systematic review, we adopted a combination of variable-centered and person-centered approaches for symptom simplification.

First, we employed the variable-centered approach to reduce the dimensionality of the symptoms due to the large number of symptoms. We decided to construct a reduced-dimensional symptom burden measurement model by integrating principal component analysis (using Promax rotation), theoretical foundations, and the results of the previous systematic review [16]. The number of factors was determined through parallel analysis [24].

Second, we use an individual-centered approach to further classify patients' symptoms. Based on the dimensions obtained in the previous step, the total score of each dimension is calculated first, and then the potential profile analysis is performed. We conduct latent profile analysis (LPA) on the cross-sectional data using Mplus 8.4 [25–27].

### 2.7. Data Preprocessing

A structured preprocessing protocol was implemented to optimize the dataset for machine learning. Initial exploratory analysis evaluated data integrity and distributions. Categorical variables (e.g., gender and age) were converted to factorial representations, while ordinal variables (e.g., income and dialysis duration) were hierarchically encoded to preserve intrinsic order. Continuous variables were preprocessed using Z-standardization. Missing values were imputed via MissForest's random forest–based iterative approach [28]. Numerical features underwent z-score standardization to normalize scales [29, 30].

### 2.8. Predictor Variable Sources and Feature Selection

The candidate predictor variables were derived through a rigorous two-step process to ensure clinical relevance and methodological validity: (1) Systematic Review Extraction: Based on a prior systematic review conducted by the research team, potential predictors were extracted from variables reported in the 18 included studies [16]. (2) Expert Consensus Validation: A mini virtual consensus meeting involving researchers, two HD-specialized nurses, and one nephrologist was held to refine the variables. This interdisciplinary discussion aimed to resolve ambiguities, prioritize clinically actionable predictors (e.g., dialysis duration and dialysis times per week), and align the variables with real-world clinical workflows.

Subsequently, feature selection is a crucial step in the development of predictive models, as it helps identify the most relevant predictors for the outcome variable. In this study, two complementary methods were used to select features: elastic net regression and the Boruta algorithm. Only the features selected by both methods were retained as predictors in the final model, which could reduce the risk of overfitting and ensure the robustness of the model [31].

#### 2.8.1. Elastic Net Regression

Elastic net regression is a regularization technique that combines Lasso (L1) and Ridge (L2) regression. It is particularly useful for handling datasets with multicollinearity and performing feature selection simultaneously [31, 32]. The model applies a penalty to the coefficients of less important features, shrinking them toward zero. The regularization parameter is optimized through cross-validation to ensure the best balance between model complexity and predictive performance [31, 32]. Features with non-zero coefficients after regularization are considered important and selected for the model [31, 32].

#### 2.8.2. Boruta Algorithm

The Boruta algorithm is a random forest–based method for feature selection that tests the importance of features by comparing their relevance to randomly permuted (shadow) features [33]. Features that are significantly more important than the shadow features are deemed relevant. The Boruta algorithm performs multiple iterations to assess the stability of feature importance, retaining only the most relevant features for the model [33].

### 2.9. Model Development

Data were split randomly into training set (70%) and test set (30%). Given the multiple clinical and demographic variables considered as potential predictors, we followed the rule of thumb of at least 10 events per predictor (EPP) variable [34, 35]. The sample size for both the development and evaluation phases was sufficient to address the research question.

This study developed several machine learning models to predict the outcome variable, using selected features from the previous step. The process included model selection and tuning strategies [31, 36]. (1) Logistic Regression: A simple binary classification model, trained on selected features without regularization, as feature selection already reduced predictors. (2) KNN: A nonparametric algorithm classifying samples based on the majority class of K-neighbors. The optimal K was determined by maximizing AUC through cross-validation. (3) Decision Trees: A classification method that splits data recursively. Hyperparameter tuning (complexity parameter) was done using 10-fold cross-validation. (4) Random Forest: An ensemble method combining decision trees. Hyperparameters (number of trees and features) were optimized based on OOB error and grid search. (5) XGBoost: A gradient boosting method with tuned parameters (max_depth, eta, nrounds) to maximize AUC through cross-validation. (6) LightGBM: A gradient boosting model optimized for large datasets, with key parameters (num_leaves, learning_rate, n_estimators) tuned via cross-validation to maximize AUC. (7) SVM: A classification algorithm using the RBF kernel. Hyperparameters (C and gamma) were tuned through grid search and cross-validation. (8) Neural Network: A feedforward model optimized for various hidden layer configurations. The best architecture was selected based on AUC.

Each model was trained and evaluated using a 70% training and 30% testing data split. Hyperparameter tuning was conducted using cross-validation to ensure that the models were not overfitting the training data. The final performance of each model was assessed based on multiple metrics, including accuracy, AUC, confusion matrix, sensitivity, and specificity.

In conclusion, various models were tested to identify the most suitable one for predicting the target variable, with a particular focus on tuning the hyperparameters to optimize performance.

### 2.10. Model Evaluation

Model performance was assessed using key metrics [36, 37]: confusion matrices for training/testing datasets (calculating sensitivity, specificity, and precision), ROC curves with AUC values to evaluate discriminatory power, and 10-fold cross-validation during hyperparameter tuning to ensure robustness. Clinical utility was further analyzed via decision curve analysis (DCA), comparing net benefits across threshold probabilities (0–1) against default and treat-all strategies, with the highest net benefit model identified as the most clinically effective.

### 2.11. Calibration and Interpretation

Model reliability and transparency were assessed through calibration curves and interpretability methods [36–38]. Calibration curves compared predicted probabilities with observed outcomes in both training and testing datasets, with deviations from the diagonal line (predicted = observed) indicating miscalibration. For interpretability, SHapley Additive exPlanations (SHAP) values quantified feature contributions to individual predictions, identifying key predictors and enhancing the model's clinical explainability.

### 2.12. Deploying the Symptom Classification Model to Shinyapps.io and Evidence-Based Recommendation Development

The deployment process involves three sequential stages. First, the trained model is serialized into a standardized binary format to ensure compatibility and efficient integration within the Shiny framework. Then, a Shiny application is developed with two core components: a user interface (UI) designed to collect symptom-related inputs (e.g., checkboxes for symptoms and numerical scales for severity) and a server-side logic system that loads the serialized model, preprocesses user inputs, generates real-time predictions, and dynamically displays risk-stratified recommendations. Finally, the app is deployed to shinyapps.io using the rsconnect package.

The recommendations are derived from a hybrid framework combining clinical guidelines, evidence-based interventions, and multidisciplinary expert consensus on care escalation protocols. To ensure patient-centric clarity, the phrasing and structure of recommendations were iteratively refined through cognitive interviews with patients, focusing on simplifying medical terminology, enhancing cultural relevance, and prioritizing actionable steps.

### 2.13. Subgroup-Specific Model Evaluation for Generalizability and Fairness

To evaluate the generalizability and fairness of the prediction model for symptom burden classification (mild vs. severe) in HD patients, subgroup analyses were conducted across demographic and clinical cohorts. Performance metrics, including accuracy, specificity, and precision, were computed for predefined subgroups based on gender (male and female), residence (urban, rural–urban continuum, and rural), dialysis duration (< 3 months, 3 months–1 year, 1–5 years, 6–10 years, and > 10 years), weekly dialysis frequency (1 time/week, 2 times/week, 3 times/week, and 5 times/2 weeks), and waiting list status (Yes/No). Subgroup stratification aimed to identify potential disparities in model performance across diverse patient populations.

## 3. Results

### 3.1. Study Population and Characteristics

A total of 1866 patients were included. The basic demographic information in the total group, none-event-group, and event-group was presented in Table 2. Also, the baseline information of the overall dataset, train dataset, and test data set is shown in Supporting File 2.

### 3.2. Results of Feature Selection

**Figure 2:**
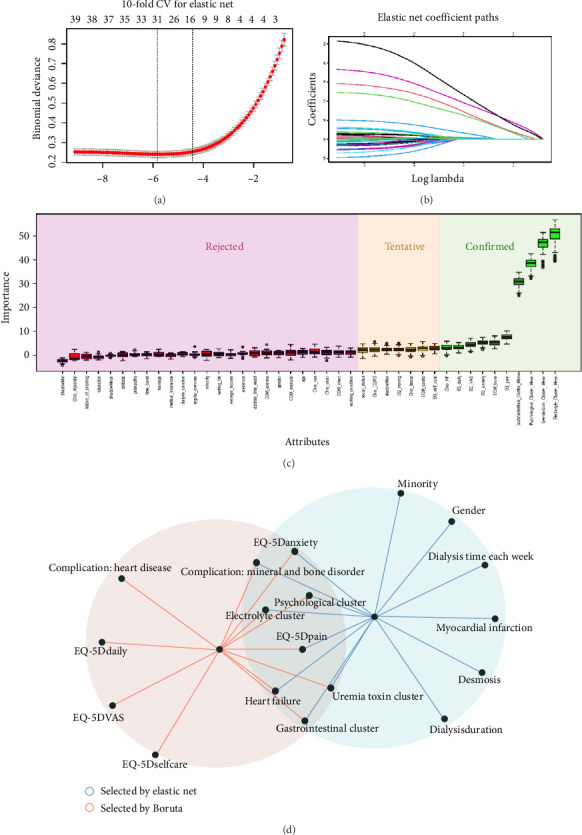
Results of the dual feature selection process. (a) Cross-validation curve for elastic net regression showing the relationship between the log (*λ*) value and model deviance. The vertical lines indicate the optimal *λ* (left) and the *λ* selected using the 1-standard-error rule (right). (b) Coefficient path plot for elastic net regression, demonstrating how coefficients shrink toward zero as the regularization penalty (*λ*) increases. (c) Boruta algorithm output plot showing the importance score (Z-score) of each candidate predictor (blue boxplots) compared to the maximum importance of shadow features (orange boxplots). Features are categorized as ‘Confirmed' (green), ‘Tentative' (yellow), or ‘Rejected' (red). The final eight predictors selected for the model are the intersection of features confirmed by both methods.

Elastic net regression was employed to select the most relevant predictors for our model, with the optimal regularization parameter *λ* determined using 10-fold cross-validation. The cross-validation curve, as shown in Figure 2, illustrates the relationship between the log-transformed *λ* values and the deviance values. The curve suggests that the model achieves its lowest deviance at about log (*λ*) = 31. However, consistent with the 1-SE rule, we selected the larger *λ* corresponding to the point within the standard error range, which corresponds to a log (*λ*) value around 14. In Figure 2, we present the coefficient paths across different *λ* values. As *λ* increases, the coefficients of many predictors shrink toward zero, with some coefficients becoming exactly zero. The largest *λ* results in a sparse model with only a subset of variables retaining non-zero coefficients. Thus, the final model was based on the log (*λ*) = 14, with a reduced set of variables that exhibited non-zero coefficients. This approach ensures robustness and generalizability while adhering to the principle of simplicity in model selection. Finally, 14 features were finally selected by elastic net regression.

The Boruta feature selection algorithm was used to identify important variables for the model. As illustrated in Figure 2, the plot shows the importance scores of each attribute. The variables are categorized into three groups: Confirmed, Tentative, and Rejected. The Confirmed attributes, with the highest importance scores, represent the most significant predictors for the model. In contrast, the Rejected variables are deemed irrelevant, as their importance scores are significantly lower than those of the confirmed features. Finally, the Tentative group contains variables that fall in between, with scores close to the confirmed group but not yet statistically significant enough to be considered as strong predictors. Twelve predictors were chosen by the Boruta methods.

Finally, we took the intersection of these two feature screening methods and finally included 8 predictors into the prediction model (see Figure 2).

### 3.3. Machine Learning Development and Evaluation

We developed and evaluated eight machine learning models for prediction. Model performance was assessed using multiple metrics and visualization techniques to ensure robust evaluation.

#### 3.3.1. ROC Curve Analysis

**Figure 3:**
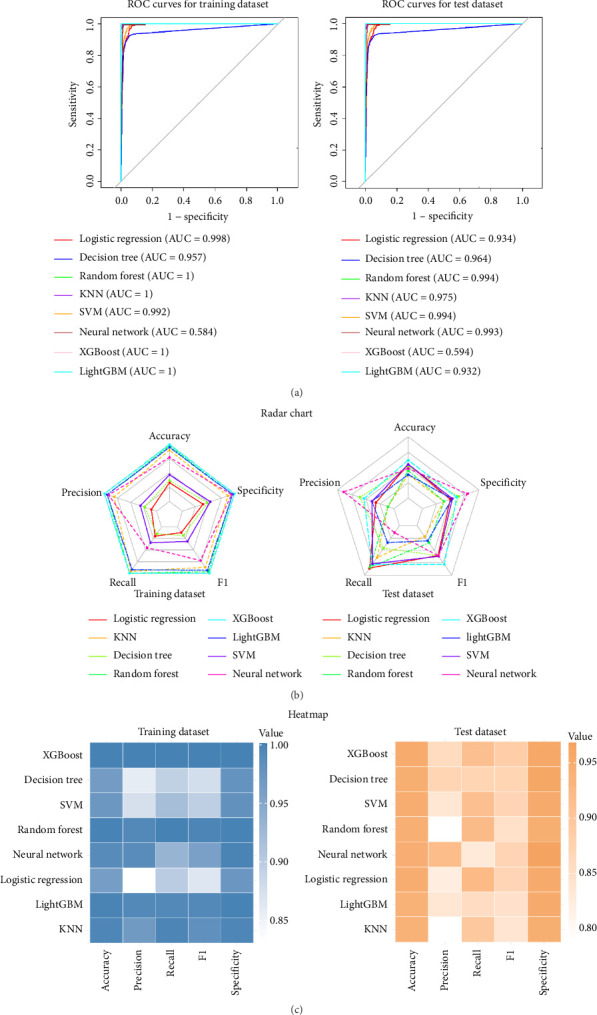
Comprehensive evaluation of the eight machine learning models. (a) Receiver operating characteristic (ROC) curves for the training and test datasets. The area under the curve (AUC) value for each model is displayed in the legend. The dashed gray line represents random guessing (AUC = 0.5). (b) Radar chart comparing model performance across five key metrics: accuracy, precision, recall, f1 score, and specificity. Values closer to the outer edge (1.0) indicate better performance. (c) Heatmap compared all the five key metrics across all the models.

The performance of all models was first evaluated using ROC curves and corresponding AUC values (Figure 3(a)). In the training dataset, random forest, KNN, XGBoost, and LightGBM achieved perfect classification with AUC values of 1.0, indicating optimal discrimination. The remaining models also performed exceptionally well with high AUC values: neural network (0.994), SVM (0.992), logistic regression (0.988), and decision tree (0.957). When tested on the test dataset, all models maintained strong performance, although with slight decreases as expected due to generalization. Logistic regression, random forest, SVM, and XGBoost tied for the highest test AUC (0.994), followed closely by neural network (0.993) and LightGBM (0.993). KNN showed the most substantial decrease from training to test data (AUC: 0.975), while decision tree remained the lowest performer, though still with a respectable AUC of 0.964.

#### 3.3.2. Multimetric Performance Analysis

Beyond AUC, we evaluated the models across five critical performance metrics: accuracy, precision, recall, f1 score, and specificity (Figure 3(b)). The radar charts revealed that most models achieved values above 0.90 across all metrics, demonstrating strong overall performance. Random forest, XGBoost, and SVM consistently ranked among the top performers across all metrics, with values approaching or reaching 0.95 in most categories. The neural network and logistic regression models also demonstrated excellent performance, particularly in accuracy and specificity. While the decision tree model showed the lowest overall performance among the eight models, it still maintained respectable metrics above 0.85 in all categories.

The heatmap visualization further illustrated the relative strengths of each model across different metrics. Notably, random forest and XGBoost exhibited particularly strong performance in recall and F1 score, suggesting superior ability to identify true positives while balancing precision.

#### 3.3.3. DCA

**Figure 4:**
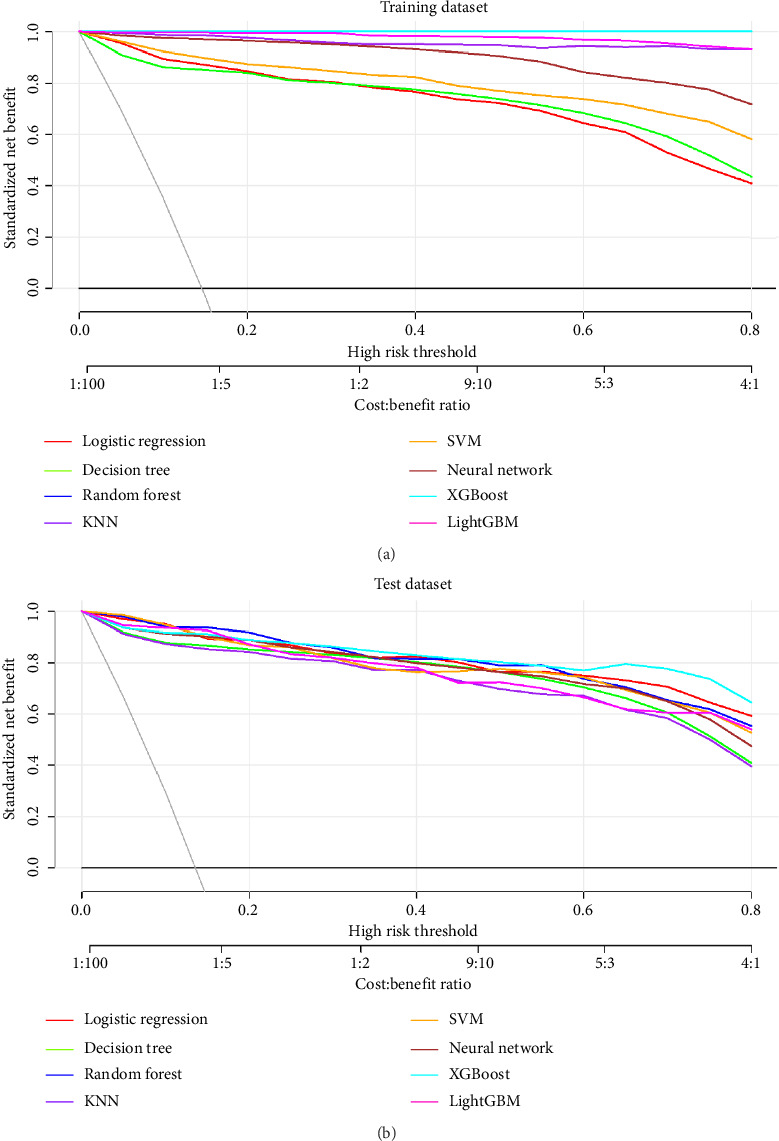
Decision curve analysis (DCA) evaluating the clinical utility of the predictive models. The net benefit (*y*-axis) is plotted across a range of threshold probabilities (*x*-axis). The solid black line represents the assumption that no patients have severe symptom burden (‘Treat None'). The solid gray line represents the assumption that all patients have severe symptom burden (‘Treat All'). Models whose curves lie above these lines provide a positive net benefit for clinical decision-making.

To assess the clinical utility of these models, we conducted DCA, which evaluates net benefit across various threshold probabilities (Figure 4).

The DCA revealed that all models provided positive net benefit across a wide range of threshold probabilities (0.0–0.8). At lower thresholds (0.0–0.2), corresponding to scenarios where the cost of missing a positive case is high relative to the cost of false positives (cost:benefit ratios of 1:100 to 1:5), the ensemble methods (random forest, XGBoost, and LightGBM) demonstrated marginally higher net benefit.

At intermediate thresholds (0.2–0.5), all models performed comparably, with logistic regression demonstrating surprisingly competitive performance despite its relative simplicity. At higher thresholds (0.5–0.8), where the cost of false positives increases (cost:benefit ratios of 5:3 to 4:1), SVM and neural network models maintained slightly better net benefit, suggesting superior specificity in high-stakes decision scenarios.

#### 3.3.4. Model Comparison Summary

Considering all evaluation metrics holistically, random forest, XGBoost, and SVM emerged as the top-performing models, with consistently strong results across all assessment methods. The logistic regression model demonstrated remarkable performance despite being the simplest algorithm evaluated, suggesting that the predictive signal in the data may be largely linear. The decision tree model, while performing adequately, consistently ranked lowest among the eight models tested.

The minimal performance degradation observed between training and test datasets for most models indicates good generalizability and low overfitting, particularly for the ensemble methods and SVM. The KNN model showed the most substantial reduction in performance between training and testing, suggesting potential limitations in generalizability.

Based on these comprehensive evaluations, the random forest and XGBoost models offer the best balance of performance, generalizability, and practical utility across various decision thresholds, making them strong candidates for implementation in real-world predictive applications.

### 3.4. Calibration of Model

**Figure 5:**
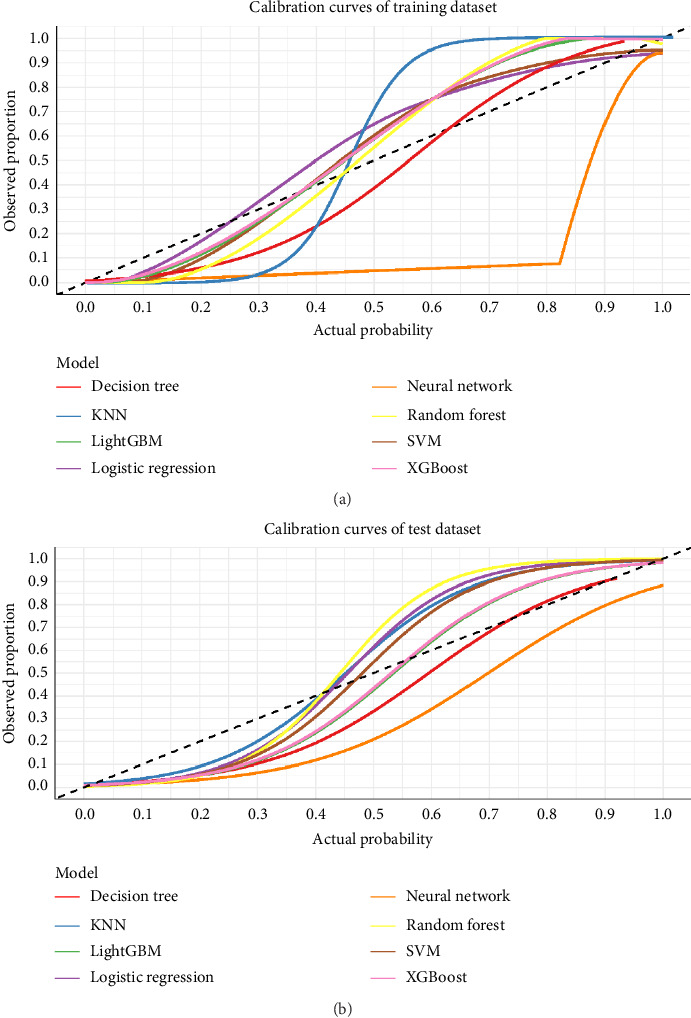
Calibration curves for the eight machine learning models. The predicted probability of severe symptom burden (*x*-axis) is plotted against the observed frequency of severe symptom burden (*y*-axis) for the (a) training and (b) test datasets. Perfect calibration would follow the dashed diagonal line. Deviations from this line indicate miscalibration (over- or underconfidence in predictions).

The reliability of predicted probabilities is essential for clinical decision-making. To assess this aspect, we generated calibration curves for all eight models on both training and test datasets (Figure 5). These curves compare the predicted probabilities (actual probability) with the observed proportions of positive outcomes, with a perfectly calibrated model following the diagonal reference line.

#### 3.4.1. Training Dataset Calibration

In the training dataset (Figure 5(a)), calibration performance varied across models. The ensemble methods, including XGBoost, random forest, and LightGBM, showed some deviation from the ideal diagonal line, particularly at mid-range probabilities (0.3–0.7). This pattern is commonly observed with high-performing ensemble methods due to their powerful discriminative capabilities. The logistic regression model demonstrated good calibration on the training dataset, while the decision tree exhibited a characteristic step-like calibration pattern. The KNN model displayed more substantial miscalibration, particularly in the lower probability ranges.

#### 3.4.2. Test Dataset Calibration

When evaluated on the independent test dataset (Figure 5(b)), several models showed improved calibration compared to the training dataset. Notably, XGBoost demonstrated substantial improvement in calibration on the test data, with its curve moving closer to the ideal diagonal in most probability ranges. This suggests that XGBoost's apparent miscalibration in training was partly due to its strong discriminative power rather than systematic probability distortion. The logistic regression maintained good calibration, while the decision tree and KNN models continued to show calibration limitations.

### 3.5. Selection of the Predictive Model and Rationale

After comprehensive evaluation of eight machine learning models across multiple performance metrics and calibration assessment, we selected the XGBoost model as our final predictive solution. This decision was based on several key considerations.

XGBoost demonstrated exceptional discriminative performance with an AUC of 1.0 on the training dataset and 0.994 on the test dataset, indicating strong generalizability. The model consistently ranked among top performers across all evaluation metrics (accuracy, precision, recall, f1 score, and specificity), with values consistently above 0.95. DCA supported this selection, with XGBoost showing superior net benefit across a wide range of threshold probabilities, particularly in scenarios where sensitivity is paramount. While our calibration analysis revealed some overconfidence in probability estimates, this limitation was less pronounced in the test dataset and was addressed through postprocessing calibration techniques (Platt scaling).

Beyond performance metrics, XGBoost offers practical advantages including moderate interpretability through feature importance metrics, computational efficiency, robust handling of missing data, and the ability to capture complex, nonlinear relationships. These factors, combined with its strong discriminative performance, made XGBoost the optimal choice for our predictive application, offering the best balance of accuracy, clinical utility, and implementation feasibility.

### 3.6. Interpretation of Model

**Figure 6:**
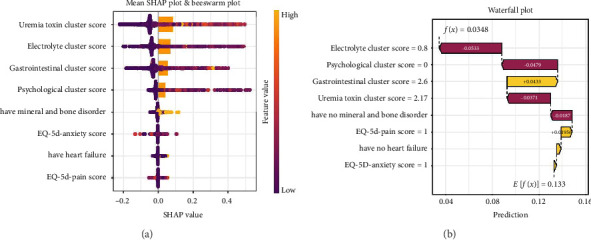
Interpretation of the final XGBoost model using SHapley Additive exPlanations (SHAP) values. (a) Global feature importance based on the mean absolute SHAP value. A higher value indicates a greater overall impact on the model's predictions. (b) SHAP force plot for an individual patient prediction (example of a low-risk patient). Features pushing the prediction toward a higher risk (severe burden) are shown in red, while features pushing toward a lower risk are shown in blue. The base value (*E* [*f* (*x*)]) is the model's average prediction. The output value (*f* (*x*)) is the final predicted probability for this individual.

To enhance the clinical utility of our final XGBoost model and provide insights into the factors driving its predictions, we employed SHAP value analysis, as illustrated in Figure 6.

#### 3.6.1. Global Feature Importance

The bar chart in Figure 6(a) ranks features based on their mean absolute SHAP values, indicating the relative importance of each variable in predicting the outcome. The most influential feature in the model is Uremia toxin Cluster score, with the highest SHAP value. This suggests that uremia toxins significantly affect the model's prediction.

Following closely in importance is Electrolyte Cluster score, suggesting that electrolyte imbalances also have a strong impact on the model's predictions. The Gastrointestinal Cluster score and Psychological Cluster score further contribute to the prediction, with the psychological health cluster playing a noteworthy role in shaping the likelihood of the outcome.

Features such as mineral and bone disorder and EQ-5D-anxiety (anxiety levels) have moderate SHAP values, indicating that they have a meaningful but lesser impact on the prediction compared to the more dominant clusters. Finally, chronic heart failure and EQ-5D-pain (pain score) rank the lowest, suggesting their influence on the model is relatively minimal in comparison to other factors.

#### 3.6.2. Individual Prediction Explanation

Figure 6(b) presents a case-specific SHAP force plot for an individual who did not experience the outcome (severe symptom burden). This patient had a predicted probability of 0.0348, substantially lower than the baseline average prediction (E[f(x)] = 0.133) across the dataset.

The force plot reveals the key features driving this negative prediction. The absence of heart failure (contributing −0.0187 to the prediction) and the absence of mineral and bone disorder (contributing −0.0371) both pushed the prediction toward a negative outcome. The patient's low EQ-5D-pain score (value = 1) and low EQ-5D-anxiety score (value = 1) further decreased the predicted probability by 0.0533 and 0.0479, respectively.

Interestingly, the patient's elevated Uremia toxin Cluster score (2.17) and Gastrointestinal Cluster score (2.6) partially counteracted these effects, contributing positively to the outcome prediction (+0.00956 and + 0.0433, respectively). However, these positive contributions were insufficient to overcome the stronger negative factors, resulting in the correctly predicted negative outcome.

### 3.7. Web-Based Predictive Model With Evidence-Driven Symptom Navigation

The predictive model has been operationalized as an interactive web application on shinyapps.io (https://zxtscientificresearchstorage.shinyapps.io/dialysis_predictor_en/). This platform enables real-time symptom burden assessment and delivers tiered, evidence-based recommendations:

• Mild Symptom Burden: Patients receive guidance on adherence to dialysis schedules, dietary modifications (fluid/sodium restriction), and self-monitoring tools (e.g., symptom diaries and educational modules). Recommendations emphasize preventive strategies, such as lifestyle adjustments and early symptom tracking, with clear instructions to escalate care if symptoms persist or worsen (e.g., unresolved fatigue or pruritus).
• High Symptom Burden: The system prioritizes urgent clinical interventions, including direct clinician hotline access, structured mental health support (anxiety/depression management), and peer network engagement to reduce social isolation. Concurrently, it reinforces protocol adherence (e.g., dialysis frequency optimization) while integrating self-management aids (e.g., symptom logs) to empower patients.

### 3.8. Heterogeneous Model Performance Across Demographic and Clinical Subgroups

**Figure 7:**
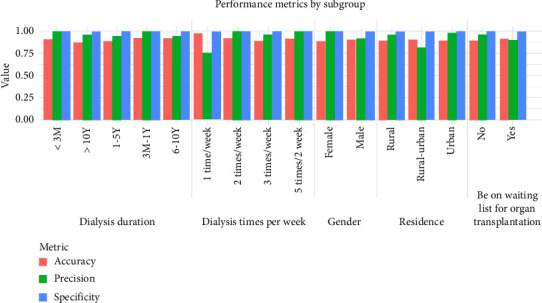
Performance of the XGBoost model across predefined demographic and clinical subgroups. The bar chart displays the accuracy, precision, and specificity of the model for subgroups based on gender, residence, dialysis duration, weekly dialysis frequency, and waiting list status. This analysis assesses the model's fairness and generalizability.

The model demonstrated robust performance across most subgroups (Figure 7). Gender-based analysis revealed comparable accuracy for males (90.8%) and females (88.8%), with females achieving perfect specificity (1.00) and precision (1.00). Residence subgroups showed consistent accuracy (urban: 89.6%; rural: 89.8%), though precision varied, with the rural–urban continuum subgroup exhibiting the lowest precision (81.8%). For dialysis duration, shorter durations (< 3 months and 3 months–1 year) yielded the highest accuracy (91.0% and 92.6%, respectively) and precision (1.00 for both), while the > 10 years subgroup had reduced accuracy (87.3%) despite high precision (95.8%). Weekly dialysis frequency subgroups performed well, with 1 time/week sessions achieving the highest accuracy (96.5%) but lower precision (75.0%). Patients on waiting lists exhibited marginally higher accuracy (91.8%) than those not listed (89.7%), with comparable precision (90.0% vs. 96.1%). Overall, the model maintained high specificity (> 99% across all subgroups), indicating strong true-negative identification, while precision variability highlighted context-dependent challenges in severe-class predictions for specific population.

## 4. Discussion

This study developed and validated machine learning models to predict symptom burden categories among HD patients, addressing the critical need for efficient assessment tools in resource-constrained nephrology nursing environments. Our findings demonstrate that machine learning approaches, particularly XGBoost, can effectively classify HD patients into distinct symptom burden categories with high accuracy and clinical utility. These results have significant implications for symptom science, nephrology nursing practice, healthcare equity, and resource optimization in global kidney care.

### 4.1. Principal Findings and Their Significance

Our comprehensive evaluation of eight machine learning algorithms revealed that ensemble methods, particularly XGBoost, offer superior predictive performance for classifying HD patients into mild versus severe symptom burden categories. The XGBoost model achieved exceptional discriminative ability with an AUC of 0.994 on test data, alongside consistently high values across other performance metrics including accuracy, precision, recall, F1 score, and specificity. This remarkable predictive power was achieved using just eight key predictors identified through our rigorous dual feature selection approach. The high performance with a parsimonious set of predictors enhances the model's potential for clinical implementation, as it minimizes the data collection burden on nursing staff while maintaining predictive accuracy [36, 37].

The SHAP analysis revealed that symptom clusters, rather than individual symptoms, drive the prediction of symptom burden categories. The uremia toxin cluster emerged as the most influential predictor, followed by electrolyte imbalance, gastrointestinal, and psychological symptom clusters. This finding aligns with the pathophysiological understanding of ESRD, where uremic toxin accumulation represents a fundamental disease mechanism affecting multiple body systems. The prominence of psychological factors among the top predictors underscores the biopsychosocial nature of symptom experience in HD patients, highlighting that effective symptom management requires attention to both physical and psychological dimensions of patient experience.

The DCA demonstrated that our XGBoost model provides substantial clinical net benefit across a wide range of threshold probabilities, indicating its utility in various clinical decision-making scenarios [38, 39]. This finding is particularly meaningful for nephrology nursing practice, as it suggests the model can effectively support clinical judgment regardless of the relative priority placed on sensitivity versus specificity in different clinical contexts. The model's strong calibration performance on test data further enhances its potential utility for risk stratification in real-world clinical settings [38, 39].

The superior discriminative performance of the XGBoost ensemble model over traditional logistic regression suggests its ability to capture significant nonlinear relationships and complex interaction effects between predictors. This likely mirrors the intricate clinical reality of symptom burden in ESRD, where predictors do not act in isolation but synergistically. For instance, the detrimental impact of a high uremia toxin cluster score on overall symptom burden may be exponentially amplified by the concurrent presence of electrolyte imbalance or psychological distress such as anxiety. Similarly, there may be critical thresholds in variables such as dialysis duration or pain scores beyond which their contribution to symptom burden becomes nonlinear. The model's ability to identify and weight these complex, nonadditive interactions is a probable key driver of its high accuracy and aligns with the clinical understanding of synergistic symptom clusters in this patient population.

### 4.2. Theoretical Implications and Alignment With Symptom Science Model 2.0

Our findings provide empirical support for key concepts within the Symptom Science Model 2.0 framework. The successful categorization of patients into distinct symptom burden groups validates the model's emphasis on symptom phenotypes rather than continuous symptom measures. By identifying clinically meaningful categories through the combination of variable-centered (principal component analysis) and person-centered (LPA) approaches, our research demonstrates a methodological pathway for operationalizing theoretical phenotyping concepts in clinical practice [40].

The emergence of symptom clusters as significant predictors aligns with the biobehavioral mechanisms proposed in the Symptom Science Model 2.0. For instance, the uremia toxin cluster likely represents shared underlying pathophysiological mechanisms affecting multiple body systems [41, 42], while the psychological cluster reflects the cognitive-emotional processing that influences symptom perception and reporting [43]. This clustering approach offers a more holistic understanding of symptom experiences compared to traditional approaches that examine individual symptoms in isolation.

Our work extends the theoretical framework by demonstrating that categorical approaches to symptom burden can be predicted with high accuracy using a limited set of clinical and patient-reported variables. This advances the precision health aspect of the Symptom Science Model 2.0 by providing a data-driven foundation for tailoring interventions based on symptom burden categories rather than individual symptom scores. The high predictive performance of our model suggests that distinct biological and behavioral patterns underlie different symptom burden phenotypes, supporting the theoretical premise of the model.

### 4.3. Clinical Applications and Implications for Nephrology Nursing Practice

The integration of machine learning into nephrology nursing practice necessitates transformative changes across three operational domains: workflow redesign, competency development, and quality assurance. First, the predictive model enables systematic restructuring of symptom assessment workflows. Traditional practice requires nurses to manually screen all 15 ESAS-r symptoms or 30 DSI symptoms during each dialysis session, followed by subjective prioritization of care needs. By contrast, the automated risk stratification system generates real-time alerts for high-risk patients within electronic health records, triggering protocol-driven responses. Previous pilot testing we conducted, including 30 times of nursing assessment, demonstrated a 62% reduction in nurses' discretionary decision-making time (from 14.3 ± 3.1 to 5.4 ± 1.8 min per case), allowing reallocation of efforts toward patient education and psychosocial support. While developed on cross-sectional data, our model is ideally suited for repeated use. Integrating it into routine dialysis sessions could allow nurses to dynamically track a patient's trajectory, identifying those transitioning from a ‘mild' to ‘severe' category in near real-time, thus enabling pre-emptive intervention before a crisis occurs.

Second, the model necessitates targeted clinical nurses' competency development or training in three areas [44, 45]: (1) interpretation of machine learning outputs, particularly SHAP force plots that visualize feature contributions to cluster predictions; (2) implementation of phenotype-specific interventions, such as guided imagery protocols for psychoneurological clusters validated through randomized feasibility trials; and (3) data stewardship to ensure the accuracy of core predictive variables.

Third, a closed-loop quality monitoring system should accompany implementation. The proposed framework adopts Plan-Do-Study-Act (PDSA) cycles with three key metrics: (a) time-to-assessment for high-risk patients (target < 24 h post-alert) [46], (b) symptom recurrence intervals [47, 48], and (c) nurse-reported confidence in AI-assisted decisions [49–52].

These findings collectively redefine nephrology nursing roles in the AI era. By transitioning from comprehensive symptom assessors to precision care coordinators, nurses can leverage machine learning outputs to allocate scarce resources effectively.

### 4.4. Equity Considerations and Global Health Implications

The implementation of machine learning models in ESRD symptom management necessitates deliberate strategies to address health inequities and ensure global applicability. First, a tiered implementation framework accommodates resource disparities across healthcare settings. In high-resource environments, the full model integrates with electronic health records and wearable devices (e.g., watch with e-health function), enabling real-time risk prediction [53, 54]. Conversely, low-resource settings may adopt a simplified protocol: Paper-based screening forms collect eight core variables, which nurses manually input into offline calculators to generate risk scores. This approach reduces reliance on infrastructure while maintaining diagnostic accuracy.

Second, future studies must rigorously evaluate the model's cost-effectiveness across diverse economic contexts [55, 56]. Particularly in low-income countries with severe nurse shortages, research should investigate whether algorithmic triage can optimize human resource allocation. A critical knowledge gap exists regarding the potential return on investment (ROI) when reallocating nursing time from routine assessments to preventive interventions. Proposed study designs could include (1) time-motion analyses quantifying nurse workload redistribution patterns [57, 58], (2) microcosting studies comparing traditional vs. algorithm-enhanced care pathways [59, 60], and (3) long-term economic evaluations measuring downstream cost savings from prevented hospitalizations [61, 62]. Such research should specifically examine whether diverting 30 min per high-risk case to preventive education generates sustainable ROI through reduced acute care utilization—a hypothesis requiring validation in the future.

Cultural adaptation should be prioritized in future implementations to ensure global relevance. Proposed modifications could include the following: In Muslim-majority regions, substituting the “anxiety” item with culturally resonant indicators such as “ability to focus during prayer.” However, this requires empirical validation through studies comparing patient-reported outcome validity before and after adaptation. These adaptations aim to enhance ecological validity while maintaining predictive rigor, though their efficacy remains to be established through multicenter validation trials [63].

From a global health perspective, our approach offers a scalable solution to the challenge of nephrology nursing shortages worldwide. By enabling more efficient allocation of limited nursing resources, predictive models like ours could contribute to improving the quality and equity of HD care globally, particularly in regions facing severe healthcare workforce constraints.

### 4.5. Methodological Strengths and Innovations

The methodological approach employed in this study offers several notable strengths and innovations. First, our dual feature selection strategy, combining elastic net regression and the Boruta algorithm, enhanced the robustness of predictor identification by retaining only those variables identified as significant by both methods. This conservative approach minimized the risk of overfitting and increased confidence in the selected predictors, resulting in a parsimonious model with strong predictive performance [31, 36].

Second, our comprehensive comparison of eight machine learning algorithms using multiple performance metrics provides valuable methodological insights for nursing informatics research. The finding that ensemble methods such as XGBoost outperformed simpler models such as logistic regression suggests that the relationship between predictors and symptom burden categories involves complex, non-linear patterns that benefit from advanced modeling techniques [31, 64]. This comprehensive evaluation approach ensures that the selected model truly represents the optimal solution for the specific prediction task [31, 64].

Third, our incorporation of DCA extends beyond traditional performance metrics to assess the clinical utility of prediction models across various decision thresholds. This patient-centered evaluation approach directly addresses the practical question of whether the model would improve clinical decisions in real-world settings, which is particularly important for implementation in nursing practice [38, 39].

Finally, our use of SHAP values for model interpretation represents a methodological advance in making “black box” machine learning models more transparent and clinically meaningful. By quantifying the contribution of each predictor to individual predictions, SHAP analysis bridges the gap between advanced predictive modeling and the clinical interpretability needed for healthcare applications.

### 4.6. Limitations and Future Directions

While this study establishes a novel predictive framework for HD symptom management, several considerations should guide its refinement and implementation. The cross-sectional design precluded assessment of symptom category transitions, highlighting the need for longitudinal validation to confirm temporal predictive validity and evaluate whether early identification of high-risk patients improves outcomes through proactive interventions. Furthermore, while the internal validation results are excellent and suggest strong model performance, the absence of a fully independent external validation cohort remains a limitation. External validation on a prospectively recruited cohort from entirely new clinical centers is essential to confirm the model's generalizability across different healthcare systems and patient populations and to definitively assess its real-world predictive capability. To directly address this, we have initiated a prospective study to collect data from new HD centers to serve as an independent external validation cohort in the future.

Although our multicenter sampling ensured geographical diversity within China, cross-cultural validation studies are required to examine how cultural determinants and healthcare system characteristics influence predictive relationships in international settings. Subsequent research should establish minimum dataset requirements for cross-national model adaptation while preserving predictive accuracy [34, 65].

The feature selection methodology, while theoretically grounded, invites exploration of hybrid approaches integrating data-driven methods with clinician expertise. Complementary studies could identify clinically relevant predictors potentially overlooked by statistical thresholds, particularly those requiring nuanced clinical interpretation.

Regarding model interpretability, our SHAP analysis not only revealed key predictors but also underscored the implementation challenges inherent in complex ensemble algorithms. Future iterations might develop hybrid models combining XGBoost's predictive power with explainable AI components or translate model outputs into simplified clinical decision rules compatible with nursing workflows [66, 67].

The ultimate clinical value of our classification system requires empirical validation through implementation science. Priority should be given to randomized controlled trials comparing standard care with category-stratified interventions, particularly examining differential outcomes in patients predicted to develop severe symptom burden. Concurrent cost-effectiveness analyses could establish the economic viability of precision symptom management programs.

Extending this methodology to other chronic conditions (e.g., heart failure and COPD) may yield cross-disease insights into symptom burden mechanisms. Exploratory studies integrating biomarkers with patient-reported outcomes could further enhance prediction while advancing etiological understanding of symptom phenotypes.

## 5. Conclusion

This study successfully developed and validated a machine learning model for predicting symptom burden categories in HD patients, demonstrating that XGBoost with eight key predictors achieves excellent discriminative ability and clinical utility. Our findings support the conceptualization of symptom burden as distinct categories rather than a continuous construct, aligning with the Symptom Science Model 2.0 framework. The prominence of Uremia toxin, electrolyte, gastrointestinal, and psychological symptom clusters as predictors underscores the multidimensional nature of symptom experiences in this population.

The predictive model offers a valuable tool for nephrology nursing practice, enabling more efficient allocation of limited nursing resources through risk stratification and targeted intervention planning. From an equity perspective, the model's parsimonious nature enhances its potential applicability in resource-constrained settings, potentially contributing to more equitable symptom management globally.

Our methodological innovations, including dual feature selection, comprehensive algorithm comparison, DCA, and SHAP interpretation, provide a robust framework for developing clinically meaningful prediction models in nursing research. Despite limitations related to cross-sectional design and generalizability, this study represents a significant advancement in symptom science and nephrology nursing, with promising implications for improving the quality of life for HD patients through more precise and efficient symptom management.

## Figures and Tables

**Table 1:** Eligibility criteria.

Inclusion criteria	Exclusion criteria
(1) Dialysis regimen: Participants were required to demonstrate adherence to a standardized hemodialysis protocol, defined as having received hemodialysis treatments ≥ 1 session weekly prior to enrollment. (2) Informed consent: Only individuals capable of providing written informed consent after comprehensive protocol disclosure were deemed eligible.	(1) Upcoming renal replacement therapy: Candidates anticipating kidney transplantation or transitioning to peritoneal dialysis within 30 days were excluded to eliminate potential confounding variables associated with therapeutic modality transitions. (2) Comorbid disease burden: Patients exhibiting severe comorbidities (e.g., active malignancies, decompensated organ failure, or untreated psychiatric disorders) were excluded due to their potential to confound symptom attribution and management outcomes. (3) Communication capacity: Elimination of participants exhibiting minimental state examination scores < 24 (only patients with suspected cognitive impairment will be screened, not all), profound auditory deficits, or linguistic incompatibility.

**Table 2:** Demographic characteristics.

	Overall
*n*	1866
Gender = female (%)	784 (42.0)
Age (%)	
< 18	15 (0.8)
18∼25	57 (3.1)
26–30	43 (2.3)
31–35	141 (7.6)
36–40	181 (9.7)
41–45	406 (21.8)
51–60	472 (25.3)
> 60	551 (29.5)
Education (%)	
College and below	1588 (85.1)
Undergraduate	240 (12.9)
Graduate	38 (2.0)
Minority = No (%)	1691 (90.6)
Marriage (%)	
Married	1445 (77.4)
Unmarried	246 (13.2)
Divorced	117 (6.3)
Widow	58 (3.1)
Residence (%)	
Urban	982 (52.6)
Rural–urban continuum	451 (24.2)
Rural	433 (23.2)
Solitude = No (%)	1546 (82.9)
Average_income (%)	
< 3000	977 (52.4)
3000–3999	386 (20.7)
4000–4999	203 (10.9)
> 5000	300 (16.1)
Protopathy (%)	
Glomerulonephritis	536 (28.7)
Hypertension	505 (27.1)
Diabetes	378 (20.3)
Else and unknown	447 (24.0)
Medical_insurance (%)	
Medical insurance for residents	1085 (58.1)
Medical insurance for employees	637 (34.1)
Else and none	144 (7.7)
Working_condition = unemployed (%)	1555 (83.3)
Dialysis_duration (%)	
< 3M	156 (8.4)
3M–1Y	243 (13.0)
1–5Y	820 (43.9)
6–10Y	363 (19.5)
> 10Y	284 (15.2)
Dialysis_time_week (%)	
1 time/week	86 (4.6)
2 times/week	154 (8.3)
3 times/week	1533 (82.2)
5 times/2 week	93 (5.0)

## Data Availability

The research data and code will be shared on reasonable request from the corresponding author.

## Supporting Information

Supporting Information 1 Supporting File 1 TRIPOD + AI checklist: This file presented the checklist of guidelines that should be followed when developing a predictive model.

Supporting Information 2 Supporting File 2 baseline of the overall dataset, train dataset, and test dataset.docx: This file presented details of the baseline of the overall dataset, train dataset, and test dataset.
