# Supplementary material for: Optimizing Nursing Communication for Symptom Management in Hemodialysis: Development of an Artificial Intelligence–Based Web Predictive Model for Burden Classification and Evidence Navigation
Source: J Nurs Manag. 2025 Oct 29;2025:4579091. doi: 10.1155/jonm/4579091 (PMC12588766; doi:10.1155/jonm/4579091)
Supplement: Supporting Information 2 — Supporting File 2 baseline of the overall dataset, train dataset, and test dataset.docx: This file presented details of the baseline of the overall dataset, train dataset, and test dataset. [file 4579091.f2.docx]

# Supplementary file 2 baseline of the overall dataset, train dataset and test data set

|  | Overall | train data set | test data set |
| --- | --- | --- | --- |
| n | 1866 | 1307 | 559 |
| Result = Yes (%) | 267(14.3%) | 191 (14.6) | 76 (13.6) |
| gender = female (%) | 784 (42.0) | 538 (41.2) | 246 (44.0) |
| age (%) |  |  |  |
| <18 | 15 (0.8) | 13 (1.0) | 2 (0.4) |
| 18~25 | 57 (3.1) | 44 (3.4) | 13 (2.3) |
| 26-30 | 43 (2.3) | 30 (2.3) | 13 (2.3) |
| 31-35 | 141 (7.6) | 101 (7.7) | 40 (7.2) |
| 36-40 | 181 (9.7) | 131 (10.0) | 50 (8.9) |
| 41-45 | 406 (21.8) | 287 (22.0) | 119 (21.3) |
| 51-60 | 472 (25.3) | 319 (24.4) | 153 (27.4) |
| ＞60 | 551 (29.5) | 382 (29.2) | 169 (30.2) |
| education (%) | |  |  |
| college and below | 1588 (85.1) | 1118 (85.5) | 470 (84.1) |
| undergraduate | 240 (12.9) | 165 (12.6) | 75 (13.4) |
| graduate | 38 (2.0) | 24 (1.8) | 14 (2.5) |
| minority = No (%) | 1691 (90.6) | 1186 (90.7) | 505 (90.3) |
| marriage (%) | |  |  |
| married | 1445 (77.4) | 1007 (77.0) | 438 (78.4) |
| unmarried | 246 (13.2) | 182 (13.9) | 64 (11.4) |
| divorced | 117 (6.3) | 84 (6.4) | 33 (5.9) |
| widow | 58 (3.1) | 34 (2.6) | 24 (4.3) |
| residence (%) | |  |  |
| urban | 982 (52.6) | 676 (51.7) | 306 (54.7) |
| rural-urban continuum | 451 (24.2) | 314 (24.0) | 137 (24.5) |
| rural | 433 (23.2) | 317 (24.3) | 116 (20.8) |
| solitude = No (%) | 1546 (82.9) | 1083 (82.9) | 463 (82.8) |
| social_status (%) | |  |  |
| 0 | 93 (5.0) | 68 (5.2) | 25 (4.5) |
| 1 | 210 (11.3) | 142 (10.9) | 68 (12.2) |
| 2 | 262 (14.0) | 177 (13.5) | 85 (15.2) |
| 3 | 365 (19.6) | 266 (20.4) | 99 (17.7) |
| 4 | 403 (21.6) | 284 (21.7) | 119 (21.3) |
| 5 | 261 (14.0) | 180 (13.8) | 81 (14.5) |
| 6 | 131 (7.0) | 93 (7.1) | 38 (6.8) |
| 7 | 141 (7.6) | 97 (7.4) | 44 (7.9) |
| average_income (%) | |  |  |
| <3000 | 977 (52.4) | 699 (53.5) | 278 (49.7) |
| 3000-3999 | 386 (20.7) | 255 (19.5) | 131 (23.4) |
| 4000-4999 | 203 (10.9) | 146 (11.2) | 57 (10.2) |
| >5000 | 300 (16.1) | 207 (15.8) | 93 (16.6) |
| history_of_smoking (%) | |  |  |
| never | 1173 (62.9) | 826 (63.2) | 347 (62.1) |
| qitted | 407 (21.8) | 278 (21.3) | 129 (23.1) |
| smoking | 286 (15.3) | 203 (15.5) | 83 (14.8) |
| regular_exercise = No (%) | 1195 (64.0) | 828 (63.4) | 367 (65.7) |
| protopathy (%) | |  |  |
| glomerulonephritis | 536 (28.7) | 378 (28.9) | 158 (28.3) |
| hypertension | 505 (27.1) | 366 (28.0) | 139 (24.9) |
| diabetes | 378 (20.3) | 259 (19.8) | 119 (21.3) |
| else and unknown | 447 (24.0) | 304 (23.3) | 143 (25.6) |
| time_travel (%) | |  |  |
| <30min | 737 (39.5) | 510 (39.0) | 227 (40.6) |
| 30-60min | 543 (29.1) | 373 (28.5) | 170 (30.4) |
| >60min | 586 (31.4) | 424 (32.4) | 162 (29.0) |
| medical_insurence (%) | |  |  |
| medical insurance for residents | 1085 (58.1) | 775 (59.3) | 310 (55.5) |
| medical insurance for employees | 637 (34.1) | 435 (33.3) | 202 (36.1) |
| else and none | 144 (7.7) | 97 (7.4) | 47 (8.4) |
| working_condition = unemployed (%) | 1555 (83.3) | 1100 (84.2) | 455 (81.4) |
| dialysis_duration (%) | |  |  |
| <3M | 156 (8.4) | 112 (8.6) | 44 (7.9) |
| 3M-1Y | 243 (13.0) | 174 (13.3) | 69 (12.3) |
| 1-5Y | 820 (43.9) | 576 (44.1) | 244 (43.6) |
| 6-10Y | 363 (19.5) | 254 (19.4) | 109 (19.5) |
| >10Y | 284 (15.2) | 191 (14.6) | 93 (16.6) |
| dialysis_time_week (%) | |  |  |
| 1 time/week | 86 (4.6) | 57 (4.4) | 29 (5.2) |
| 2 times/week | 154 (8.3) | 113 (8.6) | 41 (7.3) |
| 3 times/week | 1533 (82.2) | 1070 (81.9) | 463 (82.8) |
| 5 times/2week | 93 (5.0) | 67 (5.1) | 26 (4.7) |
| COM_cardio = Yes (%) | 883 (47.3) | 606 (46.4) | 277 (49.6) |
| COM_malnutri = Yes (%) | 272 (14.6) | 198 (15.1) | 74 (13.2) |
| COM_infect = Yes (%) | 63 (3.4) | 45 (3.4) | 18 (3.2) |
| COM_anemia = Yes (%) | 737 (39.5) | 513 (39.3) | 224 (40.1) |
| COM_bone = Yes (%) | 630 (33.8) | 452 (34.6) | 178 (31.8) |
| waiting_list = No (%) | 1684 (90.2) | 1184 (90.6) | 500 (89.4) |
| Chro_mycardio = Yes (%) | 64 (3.4) | 39 (3.0) | 25 (4.5) |
| Chro_HF = Yes (%) | 128 (6.9) | 95 (7.3) | 33 (5.9) |
| Chro_vas = Yes (%) | 165 (8.8) | 120 (9.2) | 45 (8.1) |
| Chro_tissue = Yes (%) | 92 (4.9) | 66 (5.0) | 26 (4.7) |
| Chro_ulcer = Yes (%) | 105 (5.6) | 74 (5.7) | 31 (5.5) |
| Chro_COPD = Yes (%) | 54 (2.9) | 42 (3.2) | 12 (2.1) |
| EQ_moving (%) | |  |  |
| 1 | 1319 (70.7) | 933 (71.4) | 386 (69.1) |
| 2 | 321 (17.2) | 219 (16.8) | 102 (18.2) |
| 3 | 127 (6.8) | 87 (6.7) | 40 (7.2) |
| 4 | 42 (2.3) | 31 (2.4) | 11 (2.0) |
| 5 | 57 (3.1) | 37 (2.8) | 20 (3.6) |
| EQ_self_care (%) | |  |  |
| 1 | 1533 (82.2) | 1070 (81.9) | 463 (82.8) |
| 2 | 176 (9.4) | 122 (9.3) | 54 (9.7) |
| 3 | 78 (4.2) | 58 (4.4) | 20 (3.6) |
| 4 | 29 (1.6) | 23 (1.8) | 6 (1.1) |
| 5 | 50 (2.7) | 34 (2.6) | 16 (2.9) |
| EQ_daily (%) | |  |  |
| 1 | 1407 (75.4) | 988 (75.6) | 419 (75.0) |
| 2 | 266 (14.3) | 181 (13.8) | 85 (15.2) |
| 3 | 107 (5.7) | 75 (5.7) | 32 (5.7) |
| 4 | 42 (2.3) | 29 (2.2) | 13 (2.3) |
| 5 | 44 (2.4) | 34 (2.6) | 10 (1.8) |
| EQ_pain (%) | |  |  |
| 1 | 1053 (56.4) | 738 (56.5) | 315 (56.4) |
| 2 | 621 (33.3) | 427 (32.7) | 194 (34.7) |
| 3 | 141 (7.6) | 103 (7.9) | 38 (6.8) |
| 4 | 31 (1.7) | 23 (1.8) | 8 (1.4) |
| 5 | 20 (1.1) | 16 (1.2) | 4 (0.7) |
| EQ_anxiety (%) | |  |  |
| 1 | 1099 (58.9) | 774(59.2%) | 325 (58.1%) |
| 2 | 557 (29.8) | 384(29.4%) | 173 (30.9%) |
| 3 | 147 (7.9) | 103(7.9%) | 44 (7.9%) |
| 4 | 33 (1.8) | 23(1.8%) | 10 (1.8%) |
| 5 | 30 (1.6) | 23(1.8%) | 7 (1.3%) |
| EQ_VAS (mean (SD)) | 63.72 (23.66) | 62.71 (23.86) | 66.08 (23.02) |
| Uremia.toxin_Cluster_Mean (mean (SD)) | 1.47 (2.25) | 1.46 (2.28) | 1.47 (2.18) |
| Electrolyte_Cluster_Mean (mean (SD)) | 0.64 (1.18) | 0.64 (1.20) | 0.63 (1.14) |
| Psychological_Cluster_Mean (mean (SD)) | 0.46 (1.43) | 0.46 (1.44) | 0.44 (1.42) |
| Gastrointestinal_Cluster_Mean (mean (SD)) | 0.94 (1.50) | 0.97 (1.54) | 0.89 (1.40) |
